# Life Satisfaction and Its Relationship With Personality Traits Among Medical College Students in China

**DOI:** 10.7759/cureus.57503

**Published:** 2024-04-03

**Authors:** Ling-Ling Ding, Xiao-Hua Ren, Li-Jun Zhu, Lian-Ping He, Yan Chen, Ying-Shui Yao

**Affiliations:** 1 School of Humanities and Management, Wannan Medical College, Wuhu, CHN; 2 School of Medicine, Taizhou University, Taizhou, CHN; 3 School of Public Health, Wannan Medical College, Wuhu, CHN

**Keywords:** china, university students, prevalence, personality, life satisfaction

## Abstract

Background: Life satisfaction is a comprehensive psychological index to measure a person's life quality. Previous studies have found that population sociological factors, physiological factors, psychological factors, and social factors all affect life satisfaction, but few studies have looked at the role of stable psychological factors, such as personality, in life satisfaction. Thus, this study combined previous research results and theories to study the current situation of college students' life satisfaction and its correlation with personality.

Objective: This study aims to comprehensively assess the life satisfaction levels among university students enrolled in a medical college in China, explore their correlation with various demographic factors and personality traits, identify potential areas for intervention, and provide recommendations for improving students' overall well-being and fostering the development of a positive and healthy personality.

Methods: A stratified cluster sampling method was used to select college students from a university. The questionnaire consists of general characteristics, a life satisfaction scale, and the Big Five Inventory. Descriptive statistical methods were conducted to describe the college students' life satisfaction status; an analysis of variance was performed to compare the score of life satisfaction among different demographic features; and the correlation between the score of life satisfaction and the Big Five Inventory was also analyzed.

Results: A total of 3116 subjects were included in this survey. The life satisfaction of females was higher than that of males in the dimensions of family, friends, school, and overall satisfaction (p<0.05). The life satisfaction of males in the self dimension was higher than that of females (p<0.05). The life satisfaction of different weight types had statistical significance in the life dimension (p<0.05). The life satisfaction of family, school, and overall well-being among smoking college students was lower than that of non-smoking college students (p<0.05). The life satisfaction of non-drinking college students in family, friends, life, school, and overall life satisfaction scores was higher than those of drinking college students (p<0.05). College students who get plenty of sleep a day (more than eight hours) scored higher life satisfaction scores in the self dimension than sleep-deprived college students (p<0.05). In addition to the family dimension, students taking long physical exercise breaks every day had higher life satisfaction scores in every dimension than students lacking physical exercise (p<0.05). The mean score of personality in the agreeableness and openness dimensions is the highest. Correlation analysis showed that the personality score in each dimension was positively correlated with the life satisfaction score in each dimension except for the neuroticism dimension of personality (p<0.05).

Conclusion: The life satisfaction of college students is different for different lifestyles. The student management department should pay attention to the physical and mental health of college students with low life satisfaction and further find out the reasons for the difference in life satisfaction. Meanwhile, education should be strengthened for college students and encourage them to give up smoking and alcohol; strengthen physical training; and university education should strengthen the personality cultivation of college students.

## Introduction

Life satisfaction refers to an individual's subjective evaluation of life quality based on self-set standards, which belongs to the cognitive aspect of subjective well-being [1]. Life satisfaction is the result of cognitive factors on one's own living standard, which have an important influence on one's behavior and emotions. For college students, the level of life satisfaction will affect interpersonal communication, future career orientation, and learning motivation, which has a huge impact on the development of students and social progress. With the development and progress of human society and the change in health concepts, people's understanding and demand for health have changed. With the change in health concept, the medical model has changed from a "biomedical model" to a "life-psycho-social medical model." The research on the influencing factors of college students' life satisfaction has changed from the study of physical health to the exploration of biological, psychological, social, and genetic factors. Domestic and foreign epidemiological studies on college students' life satisfaction have found that there are many factors affecting college students' life satisfaction, such as emotional fluctuations [2], weight type, weight status cognition [3], and the number of children [4]. There is also a positive correlation between medical students' healthy diet and happiness [5].

The concept of personality refers to a distinctive cognitive, affective, and behavioral pattern that is commonly understood as the amalgamation of an individual's personality traits, temperament, and other inherent attributes. Personality possesses structural characteristics, with its formation and development primarily encompassing the establishment and construction of a personal framework. In the process of personality formation and development, if affected by internal and external adverse factors, personality cannot develop healthily, it is easy to form personality defects, and it can even cause personality disorders. As a part of personality, self-esteem is positively correlated with life satisfaction [6]. A study in China found that self-esteem has a significant predictive effect on college students' life satisfaction [7]. Students in science and engineering universities with high self-esteem have higher satisfaction with their overall life, friendship, family, study, freedom, school, and environment [8]. The explicit self-esteem of college students can predict general life satisfaction [6]. Studies have found that loneliness is significantly correlated with life satisfaction and is the mediating variable in the relationship between social support and life satisfaction. However, there is little research on the relationship between life satisfaction and the personality of medical students, especially using multidimensional life satisfaction scales to explore the relationship between different dimensions of life satisfaction and personality. This study will explore the specific dimension of life satisfaction affecting personality, which is of great significance to the cultivation of healthy personalities in medical college students.

This study puts forward the following hypothesis: gender, weight, and living habits will affect college students' life satisfaction, and college students' personality characteristics will affect their life satisfaction.

## Materials and methods

Subjects

This survey adopts the method of stratified cluster sampling and selects 3126 college students at a university in southern Anhui as the research object, and it takes the form of an anonymous questionnaire survey in the class unit. Ten invalid questionnaires were removed. (The removed criteria are that the answers to the whole questionnaire are the same or the checked options are regular, and 2/3 or more of the total number of questions are missed.) There are 3116 valid questionnaires with an effective rate of 99.68.

Ethical approval

All procedures performed in studies involving human participants were in accordance with the ethical standards of the institutional and/or national research committee and with the 1964 Helsinki Declaration and its later amendments or comparable ethical standards. The study was approved by the Academic Ethics Committee of Wannan Medical College (approval number: 2023218), and written informed consent was obtained from all the participants.

Research tools

General socio-economic demographic characteristics include gender, age, height, weight, smoking and drinking, body type, etc. The types of obesity among college students were recorded according to the body fat meter.

For multidimensional students, the life satisfaction scale was developed by Huebner. This scale is widely used in many countries (such as Canada, South Korea, and Spain) to assess adolescent life satisfaction [9]. In 2013, Zhong Weiji used this scale to measure college students' life satisfaction for the first time, and the results showed that it had good validity [10]. The scale is divided into five dimensions, namely family, friends, school, life environment, and self. Each dimension represents a specific aspect of life. The number of questions varies for each dimension: seven questions for the family and self dimensions, eight questions for the school dimension, and nine questions for both the living environment and friends’ dimensions. All questions were scored on a Likert scale ranging from strongly disagree to strongly agree, with scores ranging from 1 to 6 points. Additionally, there are 10 items that are scored in reverse order. To calculate the score of each dimension, we sum up the scores of all its corresponding questions and divide them by the total number of questions in that particular dimension. General life satisfaction (overall life satisfaction) can be determined by summing up the scores of all dimensions and dividing it by 5. A higher score indicates a greater level of overall or specific life satisfaction.

The Big Five Inventory was compiled by John, Donahue, and Kentle in 1991. It contains 44 items and has good reliability and validity. The scale has five dimensions: neuroticism (N), extraversion (E), openness (O), agreeableness (A), and conscientiousness (C). Each dimension contains 8-10 items; each item adopts a Likert 5-level scoring form (1 = strongly disagree, 2 = relatively disagree, 3 = neither agree nor disagree, 4 = relatively agree, 5 = strongly agree), and 15 items are scored in reverse [11]. The lower the neuroticism score, the more stable the mood; the higher the score, the more unstable the mood; the lower the score below 20.4 is a typical low score; and the higher the score above 38.8 is a typical high score. The higher the extraversion score, the more extroverted the personality, with a score below 26 as a typical low score and a score above 42 as a typical high score. The higher the openness score, the more cheerful the personality, with a typical low score below 32 and a typical high score above 47. The higher the agreeableness score, the more agreeable the personality, with a score below 30 as a typical low score and a score above 48 as a typical high score. The higher the sense of conscientiousness score, the stronger the sense of responsibility, with a typical low score below 36 and a typical high score above 44.

Quality control

During the questionnaire design stage, as for the general population sociology data, this study selected the factors that have an impact on life satisfaction according to the results of previous studies. In addition, the questionnaire with reliability and validity verification, in line with the purpose of this study, was selected as far as possible to ensure the scientificity and validity of the study. During the test stage, in order to ensure the influence of the test stage on subjects, we printed the guide in front of the questionnaire. The investigators received unified training, and unified guiding terms were used in the investigation. After completing the filling, recall and check in time to make up for a small number of missing or wrong fillings. During the questionnaire entry stage, the collected questionnaires were collated by two people. The data was input by two people and analyzed after verification.

Data analysis

EpiData 3.2 software (EpiData Association, Denmark) was used for data entry and collation, and SPSS Statistics version 13.0 (SPSS Inc., SPSS for Windows, Version 13.0, Chicago, SPSS Inc.) was used for data analysis. Descriptive analysis was used for general demographic characteristics, and an ANOVA or t-test was used to compare the scores of various dimensions of life satisfaction among gender, weight type, exercise status, smoking, and drinking status. The correlation between life satisfaction score and personality was determined by Pearson correlation, and p<0.05 was considered statistically significant.

## Results

General demographic characteristics

There were 3116 subjects in this study, among which 1097 (35.2%) were male and 2019 (64.8%) were female. One hundred ninety-four (6.2%) were younger than or equal to 18 years old, 1080 (34.7%) were 19 years old, 1102 (35.4%) were 19 years old, 522 (17.7%) were 20 years old, and 188 (6.0%) were 20 years old or older (Table 1).

**Table 1:** Characteristics of subjects included in this study

Variables		Frequency	Percentage
Gender	Male	1097	35.20
	Female	2019	64.80
Age (years)	≤18	194	6.20
	19	1080	34.70
	20	1102	35.40
	21	552	17.70
	*≥*22	188	6.00
Weight type	Standard	1771	56.83
	Obesity	292	9.37
	Thin	318	10.20
	Invisible obesity	735	23.59
Smoking	Smoking	241	7.73
	No smoking	2875	92.27
Drinking	Drinking	1078	34.60
	No drinking	2038	65.40
Sleep time	≤8 hours	2700	86.65
	>8 hours	416	13.35
Exercise time	≤1 hour	1827	58.63
	>1 hour	1289	41.37

Comparison of life satisfaction of different genders

The results showed that females' life satisfaction scores in family, friends, school, and overall satisfaction were higher than males' (p<0.05), and males' life satisfaction scores in self-dimension were higher than females' (p<0.05) (Table 2).

**Table 2:** Comparison of various dimensions of life satisfaction between different genders

Variables	Male		Female		t	p
	Mean	SD	Mean	SD		
Family	5.03	0.72	5.16	0.63	5.53	＜0.001
Friends	4.46	0.53	4.53	0.49	3.64	＜0.001
School	4.15	0.72	4.23	0.64	3.08	＜0.001
Self	4.78	0.66	4.67	0.54	5.17	＜0.001
Life	4.16	0.67	4.2	0.64	1.72	0.09
Overall	4.51	0.49	4.56	0.42	2.72	0.01

Comparison of life satisfaction among different weight types

There were significant differences in life satisfaction among different weight types (F=3.247, p=0.021) (Table 3).

**Table 3:** Comparison of life satisfaction scores of different weight types

Variables	Weight type	Mean	SD	F	p
Family	Standard	5.11	0.65	0.568	0.636
	Obesity	5.13	0.72		
	Thin	5.08	0.67		
	Invisible obesity	5.14	0.68		
Friends	Standard	4.5	0.51	0.182	0.542
	Obesity	4.51	0.48		
	Thin	4.54	0.51		
	Invisible obesity	4.52	0.5		
School	Standard	4.21	0.66	0.919	0.431
	Obesity	4.15	0.71		
	Thin	4.17	0.67		
	Invisible obesity	4.22	0.68		
Self	Standard	4.69	0.59	1.568	0.195
	Obesity	4.72	0.62		
	Thin	4.75	0.58		
	Invisible obesity	4.73	0.59		
Life	Standard	4.16	0.63	3.247	0.021
	Obesity	4.25	0.62		
	Thin	4.14	0.69		
	Invisible obesity	4.23	0.69		
Overall	Standard	4.53	0.43	1.233	0.296
	Obesity	4.55	0.45		
	Thin	4.53	0.47		
	Invisible obesity	4.57	0.46		

Smoking and life satisfaction

The life satisfaction of smoking college students was lower than that of non-smoking college students in family, school, and overall life satisfaction (p<0.05) (Table 4).

**Table 4:** Comparison of life satisfaction scores of different smoking conditions

Variables	Smoking		No smoking		t	p
	Mean	SD	Mean	SD		
Family	4.95	0.76	5.13	0.66	3.48	＜0.001
Friends	4.49	0.54	4.51	0.5	0.65	0.52
School	4.06	0.76	4.21	0.66	2.59	0.01
Self	4.75	0.66	4.71	0.59	0.91	0.36
Life	4.1	0.63	4.19	0.65	1.76	0.08
Overall	4.47	0.48	4.55	0.44	2.05	0.04

Drinking and life satisfaction

The life satisfaction of drinking college students was lower than that of non-drinking college students in family, friends, school, life, and overall life satisfaction (p<0.05) (Table 5).

**Table 5:** Comparison of life satisfaction scores between drinking and non-drinking college students

Variables	Drinking		No drinking		t	p
	Mean	SD	Mean	SD		
Family	5.02	0.69	5.16	0.65	5.54	＜0.001
Friends	4.48	0.52	4.52	0.5	2.27	0.02
School	4.15	0.69	4.22	0.66	2.67	0.01
Self	4.72	0.63	4.7	0.58	0.78	0.44
Life	4.13	0.66	4.21	0.65	3.32	＜0.001
Overall	4.49	0.47	4.56	0.43	4.02	＜0.001

Sleep and life satisfaction

The self-dimension of life satisfaction of college students with sufficient sleep (>8 hours) was higher than that of college students with insufficient sleep (p<0.05), but there was no statistical significance in other dimensions (p>0.05) (Table 6).

**Table 6:** Comparison of life satisfaction scores at different sleep times

Variables	≤8 hours		＞8 hours		t	p
	Mean	SD	Mean	SD		
Family	5.12	0.66	5.12	0.68	0.05	0.96
Friends	4.5	0.5	4.53	0.51	1.05	0.3
School	4.21	0.67	4.18	0.68	0.99	0.32
Self	4.7	0.58	4.75	0.62	2.09	0.04
Life	4.19	0.65	4.19	0.68	0.12	0.91
Overall	4.54	0.44	4.56	0.46	1.01	0.31

Physical exercise and life satisfaction

In addition to the family dimension, the life satisfaction of college students with more than one hour of physical exercise per day was higher than that of those with insufficient physical exercise (p<0.05) (Table 7).

**Table 7:** Comparison of life satisfaction at different times of daily physical exercise

Variables	≤1 hour		＞1 hour		t	p
	Mean	SD	Mean	SD		
Family	5.11	0.68	5.13	0.64	0.7	0.48
Friends	4.49	0.5	4.54	0.5	2.86	＜0.001
School	4.16	0.67	4.26	0.66	4.06	＜0.001
Self	4.68	0.59	4.75	0.59	3.35	＜0.001
Life	4.15	0.65	4.23	0.65	3.27	＜0.001
Overall	4.52	0.44	4.58	0.44	3.65	＜0.001

Different personality dimension scores

Agreeableness and openness scored highest on average, while neuroticism scored lowest (Table 8).

**Table 8:** Personality dimension scores

Variables	Mean	SD	Minimum	Maximum
Extraversion	24.89	3.92	9.00	39.00
agreeableness	33.19	4.02	20.00	45.00
Conscientiousness	28.09	3.35	16.00	41.00
Neuroticism	22.49	4.18	8.00	38.00
Openness	33.19	4.62	14.00	50.00

Relationship between life satisfaction and personality

There was a negative correlation between neuroticism and life satisfaction (p<0.05) and a positive correlation between other dimensions of personality and life satisfaction (p<0.05) (Table 9).

**Table 9:** Correlation analysis between life satisfaction and personality dimensions Note: The data in the table are correlation coefficients r and p<0.05.

	Extraversion	Agreeableness	Conscientiousness	Neuroticism	Openness
Family	0.13	0.25	0.19	-0.22	0.16
Friends	0.27	0.37	0.19	-0.31	0.24
School	0.22	0.35	0.30	-0.30	0.24
Self	0.39	0.37	0.37	-0.35	0.47
Life	0.19	0.34	0.22	-0.29	0.20
Overall	0.32	0.46	0.35	-0.41	0.36

## Discussion

Life satisfaction between genders

Research results on the relationship between gender and life satisfaction are inconsistent, and some studies have found that there is no difference in life satisfaction between male and female students [12]. Other studies have found that the life satisfaction of female college students is higher than that of male college students [13]. The study showed that females' life satisfaction scores in family, friends, school, and overall satisfaction were higher than males' (p<0.05), and males' life satisfaction scores in self-dimension were higher than females' (p<0.05). This difference can be explained by the gender roles of men and women, which determine their different emotional experiences. Women are good at emotional expression, men seldom emphasize emotional expression, and women have more accepting attitudes than men. These differences cause their different judgments of life satisfaction, so women have a higher level of life satisfaction than men. In addition, gender roles are determined by social expectations for men and women. In traditional concepts, expectations for men are higher than expectations for women. Men have more worries about the future, while women have less pressure and higher satisfaction. However, men are more confident than women, resulting in higher self-dimension satisfaction. The gender role types of sports college students are mainly intersex and undifferentiated, and intersex students have the highest level of life satisfaction [14]. Previous studies used single life satisfaction or overall satisfaction to analyze. Multidimensional life satisfaction was used in this study, and the results showed that female college students had higher life satisfaction scores than male college students except for the life dimension. The relationship between life satisfaction and gender may be influenced by ethnic, professional, and other factors.

Weight type and life satisfaction

In this study, we evaluated the weight type of college students according to the measurement results of the body fat meter. There was no statistical significance in the scores of all dimensions of life satisfaction between different weight types except the life dimension. This result is consistent with other research results showing that the life satisfaction of college students is not related to body mass index but to the perception of weight [15]. In terms of perception of weight type, family, social, and peer evaluations also influence life satisfaction. A survey in Taizhou, China, found that there was a positive correlation between life satisfaction and obesity among men but no significant correlation between life satisfaction and obesity among women [16].

Smoking, drinking, and life satisfaction

The current situation of young students abusing addictive substances cannot be ignored. Substance abuse is the intermittent or persistent excessive use of a psychoactive substance in violation of social norms or accepted medical practice. This abuse is not simply curious use or accidental use but has a state of continuous intensification of use, which may form a heavy dependence on the substance. Substance dependence is a state of strong psychological and physical dependence after long-term abuse of a substance. Foreign studies have found that alcohol addiction is correlated with subjective well-being [17], and alcoholism [18] and drug abuse [19] are negatively correlated with life satisfaction. This study found that smoking college students had lower life satisfaction scores in the family and school dimensions and overall life satisfaction scores than those of non-smoking college students (p＜0.05); drinking college students had lower life satisfaction scores in the family, friends, school, and life dimensions than non-drinking college students (p＜0.05). The possible reason is that a good and healthy lifestyle is the basis of life satisfaction. There are few studies on the relationship between substance abuse and dependence and the life satisfaction of college students in China, and there is a lack of multidimensional studies on the life satisfaction of college students.

Sleep and life satisfaction

This study found that the self-dimension of life satisfaction of college students with sufficient sleep (＞8 hours) was higher than that of college students with insufficient sleep (p<0.05), but there was no statistical significance in other dimensions (p>0.05). The possible reason is that the factors affecting sleep quality are complex. There are many factors affecting sleep quality, such as interpersonal relationships, conflict, dormitory sleep rules, and other dormitory cultural factors, that will affect members' evaluation of the dormitory sleep environment, and the dormitory environment will have an impact on members' sleep quality [20]. An epidemiological survey found that 19.17% of medical college students showed poor quality of sleep [21]. Foreign studies have found [22] that college students' life satisfaction is related to good sleep quality. Therefore, college students should carry out mental health education, solve the problem of dormitory relationships in time, improve the sleep quality of college students, and further improve life satisfaction.

Physical exercise and life satisfaction

The meaning of life is movement. Exercise is associated with both physical and mental health, and moderate and high-intensity exercise is good for both body and mind. Studies have found a weak correlation between leisure physical activity and the life satisfaction of college students [23]. Students who regularly participate in calisthenics exercise have higher life satisfaction than non-calisthenics exercisers, and the amount of calisthenics exercise is also highly correlated with the life satisfaction of college students [24]. This study also found that, in addition to the family dimension, students taking long physical exercise breaks every day had higher life satisfaction scores in every dimension than students lacking physical exercise (p＜0.05). Students who participate in physical activity receive a range of physical, mental, and social experiences that increase their satisfaction with life. Regular physical exercise plays a positive role in maintaining college students' self-efficacy and improving their satisfaction with life. Foreign studies have also found [25] that high-intensity physical activity is positively correlated with life satisfaction. Just as the optional course of Tai Chi can improve the life satisfaction of college students [26], universities should encourage students to develop morally, intellectually, and physically, strengthen physical exercise, and promote their physical and mental health.

Personality and life satisfaction

In this study, the Big Five Inventory scale was used, and the results showed that the scores of various dimensions of life satisfaction were negatively correlated with neuroticism scores. High neuroticism scores are characterized by anxiety, depression, fear, restlessness, and emotionality [27]. The relationship between these characteristics and life satisfaction has also been verified in previous studies. Previous studies have found that college students with high levels of depression and anxiety have lower life satisfaction, and life satisfaction is negatively correlated with anxiety and depression [28]. There are also gender differences in the relationship, with female college students more likely to be depressed and anxious than their male counterparts. Another study from New Zealand came to a similar conclusion [29]. Promoting subjective well-being and improving life satisfaction can help improve the quality of life as well as the social level and academic performance of college students.

Extroversion, openness, agreeableness, and conscientiousness were all positively correlated with scores on all dimensions of life satisfaction. High scores in these dimensions belong to character strengths, which mainly refer to the positive cognition, positive feelings, and positive behaviors shown in the process of interacting with others and are reflected in the affinity of individuals in the process of interpersonal relationships, including kindness, team spirit, fairness and justice, love and being loved, sincerity, leadership, forgiveness, and gratitude [30]. At the level of consciousness orientation, college students' values and world outlook have predictive effects on life satisfaction. The results show that among optimistic students, the life satisfaction level of those with a low belief in a just world is much lower than that of those with a high belief in a just world. However, among pessimistic students, students with different levels of belief in a just world have little difference in their life satisfaction. Today's university education should pay more attention to the cultivation of college students' personalities to constantly improve their personalities.

Limitations

The scope of this study was limited to students from a single medical college, thus limiting its generalizability to the overall life satisfaction levels in medical colleges across the country. Additionally, this study did not explore variations among different academic years. In future research, it is recommended to conduct a dynamic cohort study encompassing freshmen through graduates in order to comprehensively examine the evolving dynamics of college students' life satisfaction throughout their academic journey.

## Conclusions

The life satisfaction of college students is different for different lifestyles. The student management department should pay attention to the physical and mental health of college students with low life satisfaction and further find out the reasons for the difference in life satisfaction of college students. Meanwhile, education should be strengthened for college students and encourage them to give up smoking and alcohol; strengthen physical training; and university education should strengthen the personality cultivation of college students.
